# Socioeconomical vulnerability: multiple dimensions

**DOI:** 10.1055/s-0046-1829051

**Published:** 2026-11-03

**Authors:** Leonardo C. Welling, Lis Fátima Schimiguel

**Affiliations:** 1 Universidade Estadual de Ponta Grossa, Departamento de Medicina, Ponta Grossa PR, Brazil.; 2 Universidade Estadual de Ponta Grossa, Programa de Pós-Graduação em Ciências da Saúde, Ponta Grossa PR, Brazil.

Dear Editor,

We would like to congratulate the authors of the article “Post-COVID cognitive dysfunction in socioeconomically-vulnerable older adults: findings from a Brazilian cohort.” 1 Their research highlights cognitive problems after coronavirus disease 2019 (COVID-19) in a group that is usually neglected. However, upon reading the study, some points stood out—especially the way they have dealt with socioeconomic vulnerability, which is fundamental to the entire project. They used it primarily as a participant-selection criterion, not as a determining factor in the results. Moreover, they mentioned family income and schooling, but neither was included in the final logistic regression model. Schooling proved important in the initial analysis ( *p* < 0.25) but was subsequently excluded from the models without justification.

We also noted that the definition of socioeconomic vulnerability used was quite restrictive. They only considered family income and schooling. What about other factors? Aspects such as living conditions, basic sanitation, household size, professional status, prior access to health services, food insecurity, or even race and color as indicators of inequality—none of these were considered. 2 Another point: when the authors mention that 70.8% of the participants have cognitive impairment, but do not include a control group, their interpretations become, to a large extent, conjecture. They admit that this is a limitation, but do not elaborate on how much it affects the results. Therefore, currently, it is impossible to say to what extent cognitive impairment is caused by COVID-19, or whether it stems from preexisting health problems or simply from difficult socioeconomic circumstances. The inclusion of a control group with healthy people from similar backgrounds would significantly strengthen the study's conclusions. 3

The authors point out that dementia and mild cognitive impairment are often undiagnosed in low-income communities, which is true. However, excluding participants with a history of dementia based solely on reports from family members or caregivers (without the use of tools such as the Brazilian Portuguese version of the Informant Questionnaire on Cognitive Decline in the Elderly, IQCODE) leaves a significant gap. 4 It is possible that they have included people with undiagnosed mild cognitive decline, which could inflate the number of cases of “post-COVID cognitive impairment.”

Still, we recognize the value and originality of this research. The researchers addressed an important topic and a population that is often ignored in COVID-19 studies. In the future, research should consider other aspects of socioeconomic vulnerability—housing conditions, employment status, access to healthcare, food insecurity, and race/ethnicity. And, instead of just describing socioeconomic vulnerability, studies should use it directly in their main analyses.
